# Do all roads lead to Rome? The role of neuro-immune interactions before birth in the programming of offspring obesity

**DOI:** 10.3389/fnins.2014.00455

**Published:** 2015-02-03

**Authors:** Christine L. Jasoni, Tessa R. Sanders, Dong Won Kim

**Affiliations:** Department of Anatomy, Centre for Neuroendocrinology, Gravida: National Centre for Growth and Development, University of OtagoDunedin, New Zealand

**Keywords:** inflammation, cytokines, *in utero*, brain development, DOHaD, pregnancy, epigenetics

## Abstract

The functions of the nervous system can be powerfully modulated by the immune system. Although traditionally considered to be quite separate, neuro-immune interactions are increasingly recognized as critical for both normal and pathological nervous system function in the adult. However, a growing body of information supports a critical role for neuro-immune interactions before birth, particularly in the prenatal programming of later-life neurobehavioral disease risk. This review will focus on maternal obesity, as it represents an environment of pathological immune system function during pregnancy that elevates offspring neurobehavioral disease risk. We will first delineate the normal role of the immune system during pregnancy, including the role of the placenta as both a barrier and relayer of inflammatory information between the maternal and fetal environments. This will be followed by the current exciting findings of how immuno-modulatory molecules may elevate offspring risk of neurobehavioral disease by altering brain development and, consequently, later life function. Finally, by drawing parallels with pregnancy complications other than obesity, we will suggest that aberrant immune activation, irrespective of its origin, may lead to neuro-immune interactions that otherwise would not exist in the developing brain. These interactions could conceivably derail normal brain development and/or later life function, and thereby elevate risk for obesity and other neurobehavioral disorders later in the offspring's life.

## Introduction

The period of life before birth is the most important time in our lives. During this time, developmental mechanisms put into place the cellular foundations on which our functioning, thinking, and feeling bodies will exist, in sickness and in health, across our lifespan. During the period of embryonic and fetal development, the mother's health appears to be absolutely critical to the later life health of her offspring.

This concept was first appreciated when epidemiological studies examined the long-term health outcomes of individuals who were *in utero* during the Dutch hunger winter (1944–1945). The offspring of these undernourished mothers had low birth weight and impaired glucose tolerance later in life compared to individuals born in flanking years (Ravelli et al., 1998). Interestingly, the timing of exposure to famine within the gestational period is also critically important. Thus, people exposed to famine in early gestation in the Dutch hunger winter were at greater risk of developing coronary heart disease and obesity in later life, whereas those exposed to famine in mid and late gestation were not similarly affected (Ravelli et al., 1976; Roseboom et al., 2000). Moreover, impaired glucose tolerance in offspring appeared more likely to result from undernutrition in late gestation (Ravelli et al., 1998). These observations inspired the concept that an individual's risk of disease across their lifespan could be shaped by events that occurred much earlier in their lives; indeed during their time in the womb (Barker, 2004). With the recent massive increase in obesity across the western world, much focus has now shifted to understanding the long-term health outcomes of individuals whose mothers were obese during pregnancy. Curiously, the epidemiological data paint a similar picture to undernutrition. Individuals whose mothers were obese during pregnancy show a significantly higher risk for later life obesity, and “the metabolic syndrome” (Law et al., 1992; Gale et al., 2007; Armitage et al., 2008; Crozier et al., 2010; Tamashiro and Moran, 2010; Alfaradhi and Ozanne, 2011; Ornoy, 2011). However, they also show increased risk for a constellation of behavioral and mental health problems including autism, attention deficit/hyperactivity disorder, developmental delay, anxiety, and depression (Herva et al., 2008; Rodriguez, 2010; Van Lieshout and Boyle, 2011a,b; Colman et al., 2012; Halmoy et al., 2012; Krakowiak et al., 2012; Moore et al., 2012; Wojcik et al., 2013).

Although nutrition during gestation and nutrition after birth are key contributors to neurobehavioral disease risk across the lifespan, the mechanisms that link these factors, either alone or together, to health risks and outcomes remain poorly understood. Animal models, typically rodent, sheep, or non-human primate (NHP), have been invaluable in shedding light on the cellular and molecular details occurring behind the scenes. In these models, research has traditionally focused on defining the deregulated function of offspring peripheral organs involved in metabolism, such as the pancreas, liver, adipose depots, and skeletal muscle whose function is impaired in obesity and the metabolic syndrome. However, since body weight and metabolic function are regulated by the brain (Stanley and Leibowitz, 1984; Fan et al., 1997; Cowley et al., 1999; Horvath et al., 1999; Cone et al., 2001; Elmquist et al., 2005), and since maternal nutrition during pregnancy programs an assortment of offspring neurological abnormalities, studies examining changes in the offspring brain have become increasingly prevalent.

In pregnancies complicated by maternal obesity, changes in the regions of the fetal brain that will regulate body weight later in life, including the arcuate nucleus of the hypothalamus (ARC) and the paraventricular nucleus of the hypothalamus (PVN), and brain areas associated with reward and food seeking have been reported. In the body weight regulating areas, the adult offspring of obese mothers have been reported to show: (i) altered expression of the appetite-regulating neuropeptides, including agouti-related peptide (AgRP, reduced) and pro-opiomelanocortin (POMC, reduced), in the ARC; (ii) altered expression of receptors for appetite-regulating neuropeptides, including neuropeptide Y (NPY) receptor Y1 and melanocortin receptor MC4R (both increased), in the PVN; (iii) altered neurogenesis leading to increased numbers of body weight-regulating neurons expressing orexin, melanin concentrating hormone (MCH), galanin, enkephalin, and dynorphin (Chang et al., 2008; Chen et al., 2009); (iv) ARC neuron leptin resistance, as judged by reduced pSTAT3 in response to exogenous leptin administration; (v) and developmental alterations in the neural circuitry that regulates body weight (Bouret et al., 2008; Kirk et al., 2009; Sanders et al., 2014). In the reward pathways, there are reports of altered gene expression in the opioid system (Grissom et al., 2014), changes in the mesolimbic dopamine pathways, including increased dopamine synthesis in the nucleus accumbens (NAc) and ventral tegmental area (VA), and altered dopamine responsiveness in the NAc accompanied by reduced dopamine receptor D2 expression in the VTA (Naef et al., 2008, 2011). In the offspring hippocampus, abnormalities have been observed in neural circuit formation, neurogenesis and cell death both in the late gestation fetus and in the early post-natal period (Niculescu and Lupu, 2009; Tozuka et al., 2009, 2010), increased oxidative stress, and reduced brain derived neurotrophic factor (BDNF) mRNA and protein (Tozuka et al., 2009, 2010). Such offspring also exhibited behavioral changes that are consistent with the associations between maternal obesity and some aspects of offspring mental illness observed in humans. For example, the offspring of obese non-human mothers show defects in spatial learning in the Barnes maze test (Tozuka et al., 2010), elevated anxiety (Sasaki et al., 2014), and altered reward seeking behaviors (Naef et al., 2008, 2011).

Although not comprehensive, the above summary demonstrates that substantial progress has been, and is being, made in defining the spectrum of changes that eventuate after birth when an individual has undergone gestation in an obese mother. What remains to be discovered is what happens during gestation, what molecular factors are important, and how they act on the developing organism to affect the changes that increase disease risk much later in life. Although many factors could be at work, the potential importance of the immune system stands out for two main reasons. Firstly, it has a critical and highly regulated role at the fetal-maternal interface during normal pregnancy. Secondly, pregnancies complicated by obesity, be they human or non-human, are accompanied by elevated cytokine levels in both the maternal (Catalano et al., 2009; Madan et al., 2009; Roberts et al., 2011; Kepczynska et al., 2013) and fetal circulation (Heerwagen et al., 2013; Kim et al., 2014) across gestation (summarized in Table 1). Moreover, if one considers additional complications of pregnancy that also elevate offspring risk for metabolic disease and behavioral abnormalities, one feature that they all have in common is abnormal immune system activation and elevated cytokine levels during gestation.

**Table 1:** **Summary of inflammatory response in maternal obesity across different species**.

**Species**	**Source**	**Elevated cytokines**	**Developmental stage**
**Human**	Maternal circulation	Leptin (Stewart et al., 2007; Challier et al., 2008; Catalano et al., 2009; Madan et al., 2009), CRP (Stewart et al., 2007; Challier et al., 2008; Catalano et al., 2009; Madan et al., 2009), IL6 (Challier et al., 2008; Catalano et al., 2009; Roberts et al., 2011), MCP-1 (Madan et al., 2009)	Second trimester (Madan et al., 2009), first to third trimester (Stewart et al., 2007), Pre-labor (Challier et al., 2008; Catalano et al., 2009), Term (Roberts et al., 2011)
	Placental sample (isolated placental macrophages)	IL6 (Challier et al., 2008), IL1 (Challier et al., 2008; Roberts et al., 2011), IL8 (Roberts et al., 2011), TNF (Challier et al., 2008), MCP1 (Challier et al., 2008)	Term (Challier et al., 2008; Roberts et al., 2011)
	Fetus (umbilical cord blood)	Leptin (Catalano et al., 2009), IL6 (Catalano et al., 2009)	Term (Catalano et al., 2009)
**Non-human primate**	Maternal circulation	Leptin (Frias et al., 2011)	Day 130 (of 170–180 d gestation)(Frias et al., 2011)
	Placental sample	MCP1 (Frias et al., 2011), IL1B (Frias et al., 2011)	Day 130 (of 170–180 d gestation)(Frias et al., 2011)
**Sheep**	Placental sample	TNF (Zhu et al., 2010), IL6 (Zhu et al., 2010), IL8 (Zhu et al., 2010), IL18 (Zhu et al., 2010)	Day 75 (of 135 d gestation)(Zhu et al., 2010)
**Mouse**	Maternal circulation	IL1B (Heerwagen et al., 2013; Kim et al., 2014), IL6 (Heerwagen et al., 2013; Ingvorsen et al., 2014; Kim et al., 2014), IL10 (Kim et al., 2014), IFNG (Heerwagen et al., 2013; Kim et al., 2014), TNF (Heerwagen et al., 2013; Ingvorsen et al., 2014; Kim et al., 2014), CXCL1 (Heerwagen et al., 2013), MIP2a (Heerwagen et al., 2013), CCL25 (Heerwagen et al., 2013), GM-CSF (Heerwagen et al., 2013), IL2 (Heerwagen et al., 2013), IL3 (Heerwagen et al., 2013), IL9 (Heerwagen et al., 2013)	GD15.5 (Kim et al., 2014), GD17.5 (Kim et al., 2014), GD18.5 (Heerwagen et al., 2013; Ingvorsen et al., 2014)
	Placental sample	IL6 (Kim et al., 2014), IL1b (Heerwagen et al., 2013; Kim et al., 2014), TNF (Kim et al., 2014), IL10 (Kim et al., 2014), iNOS (Heerwagen et al., 2013)	GD17.5 (Kim et al., 2014), GD18.5 (Heerwagen et al., 2013)
	Fetal circulation	IL6 (Kim et al., 2014), IL17A (Kim et al., 2014), IFNG (Kim et al., 2014)	GD17.5 (Kim et al., 2014)

## The placenta and immune system during pregnancy

There are multiple changes occurring in a mother's body during pregnancy, including increased adiposity and low-grade inflammation (Mor et al., 2011; Zhang et al., 2011). Such dynamic pregnancy-specific changes are believed, at least in part, to be responsible for the maternal adaptation to the presence of the developing fetus. Indeed, the fetus, despite being immunologically foreign, is not rejected by the maternal immune system. In mammals, this protection is partly provided by the placenta, a transient organ that forms at the interface between the mother and fetus. The placenta is composed of both maternal and fetal cells and develops upon implantation in humans and early gestation in rodents (Watson and Cross, 2005). Despite the variability in placentation across species, placental function is strikingly similar among most placental mammals (Enders and Blankenship, 1999; Malassine et al., 2003).

### Normal function of the placenta

One of the most critical functions of the placenta is to act as an immunological barrier, which protects the fetus from the maternal immune system (Kanellopoulos-Langevin et al., 2003; Mor et al., 2011). Although the full spectrum of functions that the placenta performs in its role as an immunological barrier are not well-understood, current understanding indicates that placental and decidual immune cells, such as macrophages, have an immunosuppressive function, and thus dampen the maternal immune response toward the immunologically distinct fetus (Chang et al., 1993; Lin et al., 1993; Heikkinen et al., 2003; Gustafsson et al., 2008; Houser et al., 2011; Arck and Hecher, 2013). The placenta can also respond to cytokines (Jones et al., 2009; Hsiao and Patterson, 2011); and it has been argued that this serves as a mechanism for relaying information from the maternal environment to the fetus. The ability of maternal cytokines to cross the placental barrier and directly affect the fetus is still debatable. Previous studies have shown that some cytokines, when injected into pregnant rodent dams, are able to cross the placenta and enter the fetal circulation. Some cytokines have also been shown to cross the human placenta *ex vivo* (Zaretsky et al., 2004; Dahlgren et al., 2006). This is contrasted, however, by other studies in humans and rodents, which have demonstrated a lack of cytokine transport from maternal to fetal circulation (Carbo et al., 1998; Aaltonen et al., 2005). Reconciling these datasets will require improved understanding of developmental timing in order to interweave better our knowledge of the development of cytokine-specific transport mechanisms in the placenta, changing cytokine production across gestation (Steinborn et al., 1995, 1998; Mark et al., 2013), and how both cytokine-specific and non-specific placental permeability change across gestation (Atkinson et al., 1991; Kent et al., 1994; Dahlgren et al., 2006). Nevertheless, what is clear is that the normal placenta, and its resident immune cells, performs a critical balancing act in order to be both the barrier and communicator between the mother and fetus.

### Altered placental function in maternal obesity may mean fetal exposure to cytokines and other immune system modulators

Human and animal studies have shown that maternal obesity alters placental morphology and vascularization (Hayes et al., 2012; Hayward et al., 2013; Kim et al., 2014). In rodent models of maternal obesity, decreased layer thickness, reduced trophoblast proliferation, reduced vascular supply and resultant hypoxia in the labyrinth were described (Hayes et al., 2012; Kim et al., 2014). In humans, maternal obesity caused reduced chorionic plate artery function, which may reduce blood supply to the fetus (Hayward et al., 2013). In addition, maternal obesity led to placental inflammation and altered cytokine production in human, rodent, and ovine models (Challier et al., 2008; Zhu et al., 2010; Roberts et al., 2011; Kim et al., 2014), including elevation in IL-6, IL-1β, and TNF production (all models, see Table 1 for complete list and associated animal model), as well as an increase in infiltrating monocytes and activated macrophages (Challier et al., 2008; Roberts et al., 2011; Kim et al., 2014). Also, since cytokines can alter placental nutrient transport, and maternal obesity can disrupt the placental vascular system (Jones et al., 2009; Hayes et al., 2012; Hayward et al., 2013), it is likely that fetuses developing in obese mothers would be exposed to factors from maternal and placental sources that would not be present in a normal pregnancy.

## Evidence for altered brain development and offspring obesity consequent to pathological cytokine exposure: a common link?

The idea that inappropriate exposure of the fetal brain to cytokines can interfere with neural development is an interesting idea, but has yet to gain traction in the field of developmental programming by maternal obesity, with currently only one publication suggesting that inappropriate exposure to inflammatory cytokines specifically may derail brain development (Sanders et al., 2014). Thus, we may be well-served to look to other models of developmental programming for clues to both common and diverse mechanisms leading to offspring neurobehavioral disorders. If activation of the immune system were to be a shared causative agent in the offspring programming of metabolic and mental health disorders, then any pregnancy conditions that lead to these health outcomes should also exhibit immune system activation. Below we briefly review several instances where the inflammatory environment is changed during gestation, and where adverse neurobehavioral consequences to the offspring are observed (summarized in Table 2).

**Table 2:** **Summary of different pregnancy states and their metabolic or behavioral sequelae in the offspring**.

**Maternal state**	**Offspring phenotype**	**Animal models**	**Human data**
**Maternal infection**	Neurobehavioral disorders	Maternal immune activated (MIA) rodents produce offspring with schizophrenia and ASD – like phenotypes (Smith et al., 2007; Meyer et al., 2008; Hsiao et al., 2012; Malkova et al., 2012) Offspring of IL-6 injected rats display a subset of the MIA phenotype (Smith et al., 2007)	Maternal influenza, rubella, and respiratory tract infections during pregnancy increases offspring risk for Schizophrenia and ASDs (Williams and Mackenzie, 1977; Irving et al., 2000; Smith et al., 2007; Meyer et al., 2008; Hsiao et al., 2012; Malkova et al., 2012)
	Metabolic disease	Offspring of MIA and IL-6 injected rodents display metabolic symptoms (Dahlgren et al., 2006; Pacheco-López et al., 2011) IL6 affects fetal hypothalamic circuitry development (Sanders et al., 2014)	Schizophrenic patients more likely to develop metabolic disease (Ryan et al., 2003; Thakore, 2004)
**Maternal autoimmune disease**	Neurobehavioral disorders	N/A	Maternal autoimmune diseases associated with increased risk of offspring neurodevelopmental disorders such as learning disabilities and ASD (McAllister et al., 1997; Ross et al., 2003; Neri et al., 2004)
	Metabolic disease	N/A	Maternal systemic lupus erythematosus associated with low birthweight – a risk factor for offspring obesity (Baer et al., 2011)
**Maternal smoking**	Neurobehavioral disorders	Prenatal cigarette exposure mouse model results in offspring displaying hyperactive behaviors with disrupted memory (Balsevich et al., 2014)	Maternal smoking a risk factor for offspring aggressive behavior, inattention and conduct disorder, and attention deficit disorder (Fergusson et al., 1993, 1998; Orlebeke et al., 1999; Linnet et al., 2003)
	Metabolic disease	Mice exposed to cigarette smoke during pregnancy have offspring with increased body weight and plasma leptin (Chen et al., 2011)	Maternal smoking predisposes offspring to obesity in adolescence (Power and Jefferis, 2002; Al Mamun et al., 2006)
**Air pollution exposure**	Neurobehavioral disorders	Offspring of diesel exhaust exposed mice display decreased locomotion, changes to dopamine levels, and neurodevelopmental changes (Hougaard et al., 2010; Suzuki et al., 2010; Jackson et al., 2011)	Children exposed *in utero* to pollutants from coal fired power plant had motor, language and social developmental delays (Tang et al., 2008)
	Metabolic disease	Offspring of diesel exhaust exposed mice display increased bodyweight, insulin resistance particularly on high fat diet (Bolton et al., 2012)	N/A

### Maternal immune activation

Since the late 1980s it has been acknowledged that maternal infection may play a major role in the development of offspring mental illness. Many studies have reported an association between influenza infection (as well as rubella and respiratory tract infections) during pregnancy and an increased risk for schizophrenia and autism spectrum disorders (ASD) in the offspring (Mednick et al., 1988; McGrath et al., 1994; Izumoto et al., 1999; Brown et al., 2001, 2004; Brown, 2006, 2012). However, none of these epidemiological studies in humans have been able to prove causation or suggest a mechanism beyond pointing to the link between maternal immune system activation and adverse offspring outcomes.

Because very different pathogens seem to cause the same offspring phenotype, and since it is unlikely they are able to directly infect the fetus (Williams and Mackenzie, 1977; Irving et al., 2000; Shi et al., 2005), the maternal immune response has been implicated. Maternal immune activation (MIA) rodent models have been particularly helpful to examine in more detail the *in vivo* effects of the activated maternal immune system on the developing fetal brain. These models predominantly administer either bacterial lipopolysaccharide (LPS) or double-stranded viral RNA mimic polyinosinic:polycytidylic acid (poly I:C) to pregnant rats or mice. This induces an immune response, and increases circulating levels of multiple cytokines without the confounding effects of the introduction of a viral or bacterial pathogen itself. Offspring of these MIA rodents have been reported to show a “schizophrenic-like” phenotype, including deficits in prepulse inhibition (PPI), latent inhibition (LI), anxiety, locomotion and social interaction (Smith et al., 2007; Meyer et al., 2008), as well as behaviors reminiscent of ASD (Hsiao et al., 2012; Malkova et al., 2012).

Efforts to tease apart the mechanism have found that the inflammatory cytokine IL-6 appears to play a significant role in MIA's effects on offspring behavioral programming. Inducing MIA while simultaneously blocking IL-6 actions, via IL-6 receptor deletion or IL-6 function blocking antibody, was able to prevent the ability of MIA to induce a schizophrenic-like phenotype in offspring (Smith et al., 2007). Additionally, injection of IL-6, but not the other MIA-induced cytokines IL-1α, tumor necrosis factor α (TNFα), or interferon γ (IFNγ), during pregnancy was enough to cause PPI deficits, a hallmark of schizophrenia, in offspring (Smith et al., 2007).

There is a curious link between MIA and metabolic disease, raising the possibility of some shared mechanistic underpinnings. Firstly, humans with schizophrenia appear to be more likely to develop metabolic disease (Ryan et al., 2003; Thakore, 2004; Meyer and Stahl, 2009). In addition, there is some evidence from MIA rodents that metabolic symptoms may also result from prenatal immune challenge (Pacheco-López et al., 2011). Also, injection of IL-6 into pregnant rats can produce offspring with increased adipose tissue and body weight (Dahlgren et al., 2006). Finally, IL-6 appears to affect fetal hypothalamic neural circuit development in maternal obesity (Sanders et al., 2014), and has been reported to have many neurotrophic roles (Spooren et al., 2011). Given these links, it seems plausible that inflammation, perhaps IL-6 in particular, irrespective of how it becomes elevated, impinges on fetal brain development. The big remaining questions relate to whether cytokines have their effects directly or indirectly on the brain, and/or whether they cooperate with other cytokines or abnormally elevated factors to stimulate downstream events that impinge on brain development.

### Maternal autoimmune disease

Curiously, the recent increase in obesity prevalence across the world has been mirrored by a similar rise in autoimmune disease prevalence. The literature suggests that obesity is a major contributing risk factor for the development of autoimmune disease in adults. Maternal autoimmune disease represents a spectrum of maternal disorders in which the fetus is exposed to a proinflammatory environment, and where neurobehavioral abnormalities in the offspring have been identified. For example, children whose mothers had systemic lupus erythematosus (SLE), had normal heath and intelligence scores, but a higher rate of learning disabilities such as dyslexia (McAllister et al., 1997; Ross et al., 2003; Neri et al., 2004). Additionally, primary antiphospholipid syndrome, is associated with increased risk of ASD and learning disabilities in the offspring (Nacinovich et al., 2008; Abisror et al., 2013). Although there is not yet a retrospective study examining body weight and metabolic parameters in the offspring of mothers with SLE, maternal SLE is associated with babies who are small for gestational age (Baer et al., 2011), itself a risk factor for developing obesity later in life (Barker, 2006; Gluckman et al., 2008).

### Maternal smoking

Maternal cigarette smoking, in addition to its direct effects on maternal and fetal health, predisposes offspring to increased risk of several later life adverse neurobehavioral outcomes, including aggressive behavior (Orlebeke et al., 1999), inattention and conduct disorder (Fergusson et al., 1993, 1998), and attention deficit disorder (Linnet et al., 2003). Animal models of prenatal cigarette smoke exposure have reported behavioral changes in adult offspring which parallel these human disorders (Balsevich et al., 2014). Further to this, maternal smoking is also associated with increased risk of obesity and the metabolic syndrome in the offspring, which show increased plasma leptin and impaired glucose tolerance in both humans and animal models (Power and Jefferis, 2002; Al Mamun et al., 2006; Chen et al., 2011). Although it can be difficult to tease apart the various effects of smoking during pregnancy, and exposure during early postnatal life, observations currently suggest a critical prenatal window during the first trimester of human pregnancy as the time when smoking is most likely to confer adverse health outcomes on the offspring (Toschke et al., 2003; Oken et al., 2005; Al Mamun et al., 2006).

Here, as with other complications of pregnancy that lead to developmental programming of neurobehavioral and metabolic disorders in the offspring, there appears to be chronic inflammation with elevated cytokines and other markers of immune system activation during gestation (Bermudez et al., 2002; Yanbaeva et al., 2007; Gosker et al., 2009).

### Air pollution

Human studies have found that children exposed *in utero* to pollutants from a nearby coal fired power plant had motor, language and social developmental delays (Tang et al., 2008). Animal models have shown that the air pollution present in modern day cities could cause a proinflammatory state during pregnancy, and increase the risk of metabolic and psychological disorders in the offspring. Pregnant mice exposed to common nanoparticle pollutants or diesel exhaust, showed elevated proinflammatory cytokines including IL-1β and IL-6, some of which were detected in the fetal brain (Hougaard et al., 2010; Bolton et al., 2012; Jackson et al., 2012). The offspring of mice exposed to diesel exhaust displayed decreased locomotion, changes in the levels of the neurotransmitter dopamine, and neurobehavioral changes, including a tendency to avoid the central zone in an open field test and enhanced prepulse inhibition (Hougaard et al., 2010; Suzuki et al., 2010; Jackson et al., 2011). In addition, these offspring showed increased body weight and insulin resistance, classic features of the metabolic syndrome, particularly when placed on a high-fat diet (Bolton et al., 2012).

Taking these events or exposures together with their effects on cytokine abundance and offspring outcomes, it is tempting to speculate that abnormal exposure of the fetus to immuno-modulatory molecules is the critical element linking adverse pregnancy experiences with developmental programming of obesity and neurobehavioral risks for the offspring.

## How might immuno-modulatory molecules elevate offspring risk of neurobehavioral disease?

**Figure 1:**
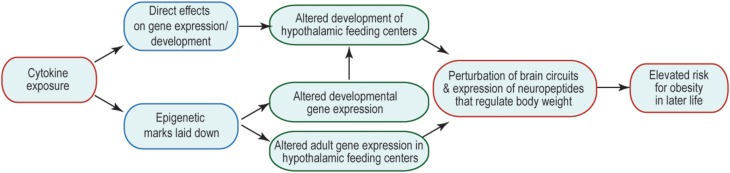
**Mechanisms by which inappropriate cytokine exposure may alter later life brain function**. Schematic indicates that cytokine exposure could result in immediate non-genomic (e.g., via kinase cascades) or genomic (e.g., change in transcription factor activity) effects on the developing brain. Cytokine exposure may also initiate activity of enzymes that catalyze epigenetic modifications, which could have immediate or long-term consequences on gene expression. The output of such cytokine exposure would be disrupted adult function, such that neural mechanisms regulating body weight do not function optimally, and thus give rise to a risk of obesity.

Many of the changes to offspring phenotype that result from prenatal cytokine exposure are likely to be underpinned by changes in brain function, including cellular physiology, neural connectivity, and gene expression. The consequences are that the offspring's neural control of body weight and behavior are perturbed and/or are less robust to environmental challenge. How prenatal exposure affects later-life function is an area of much interest, but currently very few mechanisms have been revealed to explain this curious relationship. One possibility is that prenatal brain development is altered by abnormal exposure to cytokines, leading to structural changes in the brain that might underpin suboptimal function. Another is that abnormal cytokine exposure during fetal life alters the epigenetic regulation of genes whose expression and regulation later in life is critical to appropriate body weight regulation and behavior. These options and their potential consequences to the later life ability of the brain to regulate body weight are summarized in Figure 1. Similar mechanisms might be invoked to account for other abnormal offspring behaviors, such as socialization and learning, consequent to maternal obesity; and in these cases brain areas outside hypothalamic feeding centers (e.g., those that regulate the behaviors in question) are likely to be affected.

### Access of cytokines to the fetal brain

During normal development, the embryonic and fetal brain are believed to have limited exposure to cytokines because either their circulating levels are low (early pregnancy), or because entry into the brain is regulated (mid-late pregnancy). The blood-brain barrier (BBB) is believed to become functional between GD15 and GD17 in the mouse (Ben-Zvi et al., 2014). This time is theorized to relate approximately to late second/early third trimester in the human, but the actual time of BBB formation in humans is not well-characterized. Thus, prior to BBB formation, if there were elevated cytokines in the fetal circulation they might simply diffuse into the developing brain. Later in gestation, early life and into adulthood, when the BBB is intact, the main route into the brain for blood-borne molecules is via specific transporters that move molecules, including leptin, insulin, and growth factors, from the blood into the brain (Pohl et al., 2013; Banks, 2015; Koch et al., 2014). Some molecules may also enter the brain via fenestrated capillaries in circumventricular organs or the choroid plexus. How molecules that pass through the choroid plexus endothelium and into the ventricles then pass into the brain parenchyma are not well-characterized, but transporters as well as transcellular signaling have been implicated (Balland et al., 2014). The time in development at which the genes encoding these transporters begin to be expressed is currently unknown, but abnormally elevated cytokines might gain access to the fetal brain through these normal transport mechanisms. Perturbation of either BBB formation or transporter function/gene expression in the brains of offspring developing in obese mothers are interesting further possible mechanisms that could account for abnormal exposure of the fetal brain to elevated cytokines. Another possible source of cytokines in the developing brain are microglia. These brain-resident immune cells of the monocyte lineage, are generated in the yolk sac around GD10, and can be found in the fetal brain several days later (Saijo and Glass, 2011). These seem likely candidates for producing cytokines within the brain proper, but they would need to become activated before they could do so. In support of this, recent evidence from the non-human primate suggests that microglia become abnormally activated in the prenatal hypothalamus of fetuses developing in high-fat diet-fed mothers (Grayson et al., 2010). These same fetuses also showed elevated cytokine gene expression in the hypothalamus, indicating that resident cells, such as activated microglia, provided an endogenous source of cytokines (Grayson et al., 2010).

### Direct effects of cytokines on the developing brain

Although there is no apparent role for immune system-derived cytokines in normal brain development, the activation of cytokine signal transduction pathways, including JAK-STAT and MEK/ERK, are well-known to modulate brain development, including neurogenesis, cell type specification, migration, and axon growth (Schwarting et al., 2006; Markham et al., 2007; Qin and Zhang, 2012; Lee et al., 2013; Urayama et al., 2013). Thus, should cytokines gain access to the fetal brain inappropriately, they have the potential for substantial interference with normal neural development (Figure 1). Should such a mechanism be occurring, it is predicted that individuals who were exposed to cytokines *in utero*, would show structural changes in their brains at birth. In both humans and animal models, there are numerous reports of offspring brain changes that are evident beyond the neonatal period and in adulthood. However, few studies report structural or other alterations in the brain that are evident at birth, and which are related either to pregnancy complications or later-life outcomes. On the day of birth in a mouse model of gestational obesity, the neural network regulating body weight is malformed in the offspring's brain, with fewer neural projections from the ARC reaching their targets (Sanders et al., 2014). Further exploration of the underlying mechanism revealed a role for IL-6, which altered axon growth and underlying developmental gene expression (Sanders et al., 2014). Also evident on the day of birth in a rat model of maternal obesity, were: (i) increases in the expression of orexigenic peptides, orexin and melanin concentrating hormone, in the lateral hypothalamic area; and (ii) increased galanin, enkephalin, and dynorphin expression in the PVN (Chang et al., 2008). Further exploration of the underlying mechanism revealed altered neurogenesis, leading to an increase in the generation of these cell types in the brains of fetuses developing in obese mothers (Chang et al., 2008). Also in a rat model of maternal obesity, CD11b and TLR4, both markers of activated microglia, were elevated on the day of birth (Bilbo and Tsang, 2010). This further suggests the possibility of an inflammatory state during the prenatal period. Better defining brain abnormalities as having their origins during prenatal or postnatal development (or some elements of each) will be critical to understanding underlying mechanisms, and ongoing research is beginning to provide this key information.

### Effects of cytokines on epigenetic regulators

Another possibility is that genes whose expression is critical for later life neural function are epigenetically modified at the time of cytokine exposure. Such genes may not necessarily be expressed during gestation, but the epigenetic marks laid down before birth would be there to affect gene expression whenever such genes were needed for normal neural function later in life; should such changes alter normal neural function, disease risk would be elevated. There is no need for the two mechanisms to be mutually exclusive (or exclusive of other possibilities), so it is likely that multiple mechanisms are at play when a fetus develops in an environment complicated by inappropriate cytokine exposure (Figure 1).

In many cases where life experience is associated with phenotypic change, alterations in DNA methylation, histone modifications, regulatory RNAs or some combination thereof have been observed in multiple offspring tissues. What we currently do not understand in relation to maternal obesity is: which environmental factors are important modulators of the molecules that execute epigenetic changes; how particular genes become targeted for epigenetic change by certain factors; and how a constellation of epigenetic modifications occurring across the genome affect gene function at discrete loci and at particular times during development and adult life.

Should cytokines be causative agents in developmental programming via an epigenetic mechanism, then they should be able to modulate the activity of the proteins that enact epigenetic change, but how they might do so is less clear. It is known from cancer studies that inflammation-induced DNA methylation and histone changes are associated with polycomb-group target genes, which are significant here because their regulation relies on chromatin remodeling (Hahn et al., 2008). Also in cancer cell lines, IL-6 is able to affect both the expression and nuclear translocation of DNA methyltransferase 1 (DNMT1), consequently changing its activity within the cell (Hodge et al., 2001, 2007; Foran et al., 2010), and potentially targeting methylation to specific gene promoter regions (Li et al., 2012). As many epigenetic modifying enzymes are regulated by phosphorylation cascades (Jeltsch and Jurkowska, 2014), and since cytokines are known to stimulate multiple kinase cascades, including ERK, Jnk, PI3 kinase/Akt (Hirano et al., 1997; Mak and Yeh, 2002; Pestka et al., 2004a,b), the mechanistic links seem apparent, and available for further testing. In addition, transcription factors play a key role in directing epigenetic changes to particular genomic regions (Ding et al., 2008; Hashimoto et al., 2010; Feldmann et al., 2013). Cytokines also notoriously signal through the JAK/STAT pathway, which leads to DNA binding and transcriptional modulation by the activated STAT proteins (Murray, 2007). STAT proteins already have been described for their ability to recruit DNA binding and transcriptional regulatory molecules to specific DNA sequences, so this represents another signaling pathway through which cytokine exposure could lead to epigenetic modifications across the genome (Vahedi et al., 2012; Hedrich et al., 2014; Li et al., 2014).

## Can anti-inflammation treatment protect the offspring brain?

If abnormally elevated inflammatory mediators play a causative role in the acquisition of offspring phenotype, then ameliorating them should protect normal offspring physiology. This line of reasoning has been used to test the efficacy of a number of pharmacological and physiological strategies that reduce inflammation. The literature here is growing rapidly, and this section is not intended to be exhaustive, but rather to give a glimpse into where the field is headed, and to bolster the hypothesis that increased exposure of the developing brain to inflammatory mediators is a key player in the developmental programming of offspring neurobehavioral disorders, including deregulated body weight control.

Transgenic elevation of the anti-inflammatory n-3 polyunsaturated fatty acids (PUFA) relative to the pro-inflammatory n-6-PUFAs in a mouse model of maternal obesity, reduced the metabolic phenotype of offspring, including normalizing liver triglycerides and insulin sensitivity, and reducing body weight (Heerwagen et al., 2013). Unfortunately, inflammatory markers were not examined in the brain. As the transgene would presumably have been active during development, then it is likely that an anti-inflammatory milieu during gestation played a key role in determining the offspring phenotype, however, this was not examined directly. Several other agents with broad effects including anti-inflammatory, such as resveratrol (Roberts et al., 2014) and taurine (Li et al., 2013), have also been trialed in non-human primate or rat models of maternal obesity, respectively. Both reported mixed effects and confounding details that limit their practical utility, and neither examined inflammation in the brain.

Manipulation of diet and exercise also have been used to target inflammation as a means to combat offspring consequences of maternal obesity. Reversing maternal diet from high fat to control during the lactation period and after weaning in a mouse model of maternal obesity, was able to reverse social deficits and brain inflammation in offspring (Kang et al., 2014). This suggests that the neural circuits regulating social behavior rely on a constant exposure, particularly in the neonatal period, to inflammatory mediators in order to form and/or function improperly later in life. At least some of the neural circuits governing social behaviors show a great deal of plasticity in the early postnatal period, suggesting that once inflammation is reduced, these brain areas have the ability to form/reform normally. Other brain areas, governing other behaviors, will likely have different schedules of development and plasticity. Identifying these opportune times for plasticity will be critical to applying anti-inflammatory interventions to reduce offspring metabolic disease.

Exercise is another intervention that has been used to reverse the effects of maternal obesity on offspring health. Although exercise has been used to combat a variety of health disorders, the mechanisms underlying its effects are not well-understood. In a rat model of maternal obesity, several studies have observed that exercise, in either the early postnatal period or adulthood, was able to improve the offspring metabolic dysfunction caused by maternal obesity (Bahari et al., 2013; Rajia et al., 2013). The effects of early life voluntary exercise have not examined inflammatory marker expression in the brain (Rajia et al., 2013). Exercise in the adult offspring of obese dams who received either control or high fat diet after weaning showed a trend toward reduced IL-6 mRNA levels in the ARC and IL-1β in the hippocampus following exercise compared to animals with similar diet history, but no exercise (Bahari et al., 2013).

## Conclusions

Although the causative factors that link pregnancy complications or exposures to offspring health and disease risks later in life have yet to be fully cataloged, cytokines, classically known for their roles in immune system modulation, are surfacing as key players. Doubtless there are many more factors remaining to be identified. In addition, the mechanisms by which such factors impinge on the developing fetal brain and other organs remain very poorly explored at present. Identifying these mechanisms and linking them causally with later life disease susceptibility will be a monumental challenge. Such understanding, however, is critical to developing therapeutic approaches to protect the developing brain in the face of adverse pregnancy complications. Of important further consideration, is the role of factors, including those from the immune system, in the postnatal developmental period, where the brain's extensive plasticity could be harnessed. The body of literature in this area also is growing at a rapid pace, and the interplay among prenatal and postnatal factors, immune activation, and brain development and function later in life are well on their way to being more clearly defined. The next great challenges will be to translate this mechanistic understanding into preventative and directed interventions aimed at reducing the burden of disease risk that stems from pregnancy complications and exposures that are not currently easily preventable.

### Conflict of interest statement

The authors declare that the research was conducted in the absence of any commercial or financial relationships that could be construed as a potential conflict of interest.
